# An Accurate Method for Studying Individual Microbial Lag: Experiments and Computations

**DOI:** 10.3389/fmicb.2021.725499

**Published:** 2021-11-04

**Authors:** Simen Akkermans, Jan F. M. Van Impe

**Affiliations:** ^1^BioTeC, Chemical and Biochemical Process Technology and Control, Department of Chemical Engineering, KU Leuven, Ghent, Belgium; ^2^Optimization in Engineering Center-of-Excellence (OPTEC), KU Leuven, Leuven, Belgium; ^3^Flemish Cluster Predictive Microbiology in Foods (CPMF2), Ghent, Belgium

**Keywords:** individual lag, method development, maximum likelihood estimation, Monte Carlo simulation, *Escherichia coli*

## Abstract

Variability in the behavior of microbial foodborne pathogens and spoilers causes difficulties in predicting the safety and quality of food products during their shelf life. Therefore, the quantification of the individual microbial lag phase distribution is of high relevance to the field of quantitative microbial risk assessment. To construct models that predict the effect of changes in environmental conditions on the individual lag, an accurate determination of these distributions is required. Therefore, the current research focuses on the development of an experimental and computational method for accurate determination of individual lag phase distribution. The experimental method is unique in the sense that full liquid volumes are sampled without using dilutions to detect the final population, thereby minimizing experimental errors. Moreover, the method does not aim at the isolation of single cells but at a low number of cells. The fact that several cells can be present in the initial samples instead of having a single cell is considered by the computational method. This method relies on Monte Carlo simulation to predict the individual lag phase distribution for a given set of distribution parameters and maximum likelihood estimation to find the parameters that describe the experimental data best. The method was validated both through simulation and experiments and was found to deliver a desired accuracy.

## Introduction

Microbial risks associated with the consumption of food products are studied using quantitative microbial risk assessments. These risk assessments rely on mathematical models from the field of predictive microbiology to simulate microbial behavior. The risk that undesired events will take place is calculate by including different source of variation in these model predictions through techniques such as Monte Carlo simulation. The variation comes from several different sources, e.g., experimental uncertainty and variability in processing conditions. Another important source of variability comes from the microorganism, which includes the variability in the lag phase duration (Lianou and Koutsoumanis, 2013). The lag phase duration and variability in a microbial population is a result of the lag phase duration and variability of its individuals. Therefore, the characterization of the variability in the individual lag phase duration under various environmental stresses is of high importance for improving microbial food safety and quality (Guillier et al., 2005). Future research could aim to construct mathematical models that describe the effect of changes in environmental conditions on the individual lag phase distribution. Therefore, accurate approximations of these distributions are required. The current research deals with the development and validation of an experimental and computational method for the accurate determination of the individual lag phase distribution.

Studying process variability requires a large amount of experimental data. Therefore, researchers often turn to high-throughput methods, such as optical density (OD) measurements, for studying microbial variability (Francois et al., 2005; D’Arrigo et al., 2006; Dupont and Augustin, 2009; Stringer et al., 2011; Aguirre et al., 2013; Xu et al., 2015). However, this technique comes with some limitations. First, the detection limit is high, at about 10^6^–10^7^ CFU/mL for bacterial cells (Baka et al., 2014). This means that measurements are taken close to the stationary phase and require a long period of growth when starting from low inoculum sizes. Secondly, the relationship between the OD and the true number of viable cells is influenced by the environmental conditions that the cells are subjected to. As such, using calibration curves between these measures reduces the method’s accuracy (Métris et al., 2006). Finally, there are several common practical issues when working with the microplates that are needed for these studies. They often suffer from problems with respect to evaporation of the liquid from the plate and condensation on the lid of the plates (Brewster, 2003; Walzl et al., 2012). Other high throughput methods are available, such as the analysis of time laps microscopy imaging or the ScanLag system (Pin and Baranyi, 2006; Levin-Reisman et al., 2010). However, these methods are not frequently used since they require equipment that is too expensive or not available for many labs.

Based on the downsides and difficulties with respect to OD methods, the first criterion was that the new method should work with viable plate counts. Plate count methods deliver a great accuracy by using a direct measurement of the number of living microorganisms in a sample. The downside of this technique is the high experimental load. Moreover, when considering the inoculation methods that are commonly used to isolate individual cells, the yield of experimental data over the total number of samples is relatively low. This is because most samples either contain no cells at all or have to be discarded for likely containing too many cells. The first goal will be therefore to select the most suitable inoculation method out of two conventional methods.

Two methods are commonly applied to isolate single cells. In the first method, an inoculum is serially diluted in a microplate such that the chance of achieving single cells in the last columns is maximized (Francois et al., 2003). In the other method, a solution is diluted to such a low concentration that, when distributed over a microplate, the chance of isolating single cells is maximized (Robinson et al., 2001). Both methods have a few things in common. It is inevitable that some of the samples may contain more than 1 cell. However, the computational methods that are typically used to process the data from these experiments assume that there is only a single cell in each sample that contains cells. Therefore, to increase the chance of obtaining single cells as opposed to multiple cells, the inoculum concentration should be low. A low inoculum concentration will also increase the chance of having no cells at all and therefore decreases the yield of experimental data. As such, a trade of exists between the quantity of experimental data and the quality of that data (quality being a high likelihood of having a single cell in the samples containing cells). Therefore, a computational method is required that considers the initial distribution of the population size in the calculation of the distribution of the individual cell lag. Baranyi et al. (2009) published a methodology that takes the initial concentration into account based on the method of moments. However, this method does not allow to determine the accuracy of the estimation results. In contrast, maximum likelihood estimation allows the calculation of confidence bounds on distribution parameters, which is of high importance in a modeling context. The second goal is therefore the development of a suitable computational method and comparison with the existing moments-based method.

The overall goal of this work is the development of an experimental and computational method that allows the accurate determination of the distribution of the individual cell lag. The experimental method will be designed in such a way that experimental errors are decreased to an absolute minimum by analyzing the total cell content of samples without dilutions. The computational method will be developed so that there is no longer a need to aim for single cell isolation in the experimental method, but rather to include the initial cell distribution in an accurate description of the uncertainty propagation.

## Materials and Methods

### Bacterial Strain

*Escherichia coli* K12 MG1655 (CGSC#6300) was acquired from the *E. coli* Genetic Stock Center at Yale University. A stock culture was stored at −80°C in Brain Hearth Infusion broth (BHI, Oxoid, Hampshire, United Kingdom), supplemented with 20 % (w/v) glycerol (Acros Organics, Geel, Belgium). This strain was used for all microbiological experiments in this study.

### Inoculum Preparation

The inoculum was prepared in a three step procedure: (i) A loop (10 μL) of the stock culture was spread onto a BHI agar plate, (BHIA, containing 14 g/L technical agar, VWR, Radnor, PA, United States) and incubated overnight at 37°C. (ii) Then, a single colony was transferred to a 50 mL Erlenmeyer containing 20 mL BHI broth and stored at 37°C for 9 h. (iii) Finally, 20 μL of the stationary phase culture was inoculated in 20 mL of fresh BHI broth and incubated at 37°C for 17 h.

### Detection Limit

The detection limit of the optical density (OD) measurement was defined as three time the standard deviation on the mean of a repeated measurement. The OD was measured at 595 nm using a FilterMax F5 microplate reader (Molecular Devices). The OD of a 1/8 dilution of the second preculture, was measured 24 times at a volume of 150 μL per well.

### Calibration Curve

The method used to obtain the calibration curve was based on Francois et al. (2003). All wells of a 96-well microplate were filled with 150 μL of BHI broth, except for the first column. The first and last well of the first column were filled with 300 μL of BHI solution. These will cause the first and last row to serve as blanks. The remaining 6 wells of the first column were filled 300 μL of the second preculture, after mixing it thoroughly. A 1:2 dilution series was made by transferring 150 μL from all wells of the first column to all wells of the second column. This process is repeated 10 times until 150 μL is taken from the last column and discarded. After measuring the OD, one sample of each dilution was decimally diluted and plated on BHIA plates for enumeration. BHIA plates were incubated overnight at 37°C. This entire process was performed in duplicate. A linear regression was applied to the natural logarithm of the cell density vs. the natural logarithm of the OD, yielding the following equation:


(1) ln(N)=a+b⋅ln(OD)

with N [CFU/mL] the cell density, OD [−] the OD and a and b the linear regression parameters. Given the low variance on the measurements of OD compared to viable plate counts, the former variance was considered negligible and the parameters *a* and *b* were estimated using a simple least squares regression. More details on the regression and calculation of uncertainty on the parameters and predictions is provided in section “Parameter Estimations.”

### Serial Dilution Inoculation Method

This method is based on the work of Francois et al. (2003) and aims at obtaining single cells in the last four or five rows of a microplate after serial dilutions. The concentration of the preculture was measured at 595 nm using six 150 μL samples of a 1:10 dilution of the preculture in BHI broth, using a FilterMax F5 microplate reader. The OD of the cells in these samples was calculated by subtracting the OD of six 150 μL samples of pure BHI solution. The concentration of viable cells (CFU/mL) was calculated using the calibration curve that is described in section “Calibration Curve.” Based on this calculated cell density, the preculture was further diluted to obtain 100 CFU per well containing 150 μL. Therefore, the preculture was first serially decimal diluted in BHI broth and a final dilution was made by transferring the appropriate volume of diluted preculture to 8 mL of BHI broth in a 15 mL centrifuge tube. The diluted culture (300 μL) was transferred from the Falcon tube to each well of the first row of a microplate. The remaining wells were filled with 150 μL of BHI broth each. Using a multichannel pipette, 150 μL was transferred from each well of the first to the second row of the microplate and mixed. This process was repeated for the remaining rows and 150 μL was discarded from each well of the last row. This process resulted in a 1:2 dilution series throughout the rows of the microplate with the following expected number of cells per well: 100.00, 50.00, 25.00, 12.50, 6.25, 3.13, 1.56, 0.78, 0.39, 0.20, 0.10, and 0.10 CFU.

### Low Cell Density Inoculation Method

An alternative method for the inoculation of single cells in a microplate was based on the inoculation of the entire plate with the same concentration of diluted preculture. The concentration is typically chosen below one CFU per well to have a high likelihood of obtaining single cells in those wells that contain cells. To initiate this method, the cell density of the preculture was measured and calculated in the same way as described in section “Serial Dilution Inoculation Method.” Then, the appropriate volume to achieve the desired final concentration was transferred from a serial decimal dilution of the preculture to 40 mL of BHI broth in a 50 mL centrifuge tube. Using a multichannel pipette, 150 μL of this solution was placed in every well of a microplate.

### Experimental Study of the Population Variability

Starting from streak plates, three sets of preculture Erlenmeyer’s were prepared the day before the experiments were inoculated. When diluting the preculture into the final concentration, cells were transferred in BHI broth containing 56 g/L NaCl to induce a lag phase. Three sets of 96-well plates were inoculated in the morning at different intended population sizes: (i) 4 96-well plates at 0.48 CFU/well, (ii) 4 96-well plates at 4.80 CFU/well and (iii) 2 96-well plates at 48.00 CFU/well. The method used for inoculation was the low cell density method explained in section “Low Cell Density Inoculation Method” and all wells were inoculated with a volume of 150 μL. The time of inoculation of each plate was noted down. After inoculation, all plates were sealed with a polyester microplate sealing film before closing the lid. The decimal dilution series that was used to inoculate the BHI and salt broth was also used to plate 9 drops of 20 μL of the 6th decimal dilution of the preculture for the calculation of the concentration of cells in the BHI and salt broth. The first two sets of plates were incubated overnight at 25°C. Then, all samples were transferred entirely to individual BHI agar plates that had been left to dry at atmospheric conditions for several days to allow fast absorption of the liquid and fixation of the individual cells on the agar surface. Before transferring the samples to the agar plates, they were pipetted up and down several times to make sure all cells were in suspension. The time of sampling was noted down for each sample. These plates were incubated overnight at 37°C. The number of colonies on these plates represented exactly the number of cells in the population that had developed from the low initial number of cells. The third set of plates was used to quantify the exponential growth rate of the population under identical conditions as those used for the low inoculum levels. After inoculation, three entire samples were taken and plated at three time points to be able to quantify the initial population density. During the following days, 50 μL samples were taken, diluted and plated every hour in triplicate during working hours. As such, the full growth curve from lag until stationary phase was included in the data.

### Two- and Three-Phase Linear Model

To determine the lag phase duration and maximum specific growth rate of experimental or simulation data, the following simple three-phase linear model was used:


(2) $$\begin{array}{c} \text{ln}\left(\text{N}\left(t\right)\right)=\\ {\text{N}}_{0}+\mu \cdot \left(t-\lambda \right)\cdot 1\left(\text{t}-\lambda \right)-\mu \cdot \left(\text{t}-{\text{t}}_{\max}\right)\cdot 1\left(\text{t}-{\text{t}}_{\max}\right) \end{array}$$

In this equation N is the number of cells at time t, N_0_ is the initial number of cells, is the exponential growth rate, is the lag phase duration of the population and t_max_ is the time at which the maximum population density is obtained. The function 1(⋅) represents the unit step function, which is 0 for all arguments smaller than 0 and 1 for all arguments greater or equal than 0. If no stationary phase behavior was included in the data, the last term of this equation was omitted. This will be referred to as the two-phase linear model. This equation was based on the work of Buchanan et al. (1997), (McKellar and Lu, 2004).

### Monte Carlo Simulations

Monte Carlo simulation was used in this research. Data were generated with the function *random* of MATLAB 9.7 (MathWorks), according to the appropriate distribution. The number of iterations varied depending on the desired accuracy or the experimental system that was described by the simulation. The number of iterations when describing inoculations according to the Poisson [*Pois*(*cell concentration* ⋅ *sample volume*)] distribution is expressed as the number of 96-well plates, in equivalence with the experimental methods represented by these simulations.

### Parameter Estimations

Parameter estimations were carried out based on least squares regressions using the *lsqnonlin* routine of the optimization toolbox of MATLAB 9.7. Probability density functions were fitted to data using the *fitdist* function of the same software. The method for calculating the uncertainty on the model parameters and output with the linear approximation is described in Akkermans et al. (2018).

## Results and Discussion

The current research aims to determine the parameters of the probability distribution of the individual cell lag, based on the measurement of the population density after a certain period of growth. Specifically, the method should deliver a high accuracy while requiring a reasonable experimental effort and being applicable in most microbiological labs. Based on the explanation in the introduction, it was decided that a sample inoculation method had to be selected that provides a high yield of experimental data. Computational and experimental comparison of two inoculation methods is presented in section “Comparison of Conventional Inoculation Methods.” Section “Detection Limit and Calibration Curve” will first present the results of the determination of the detection limit and calibration curve, which are essential for each of the inoculation methods. The computational method to process the experimental data requires a mathematical model that describes the stochastic process of cell division in the lag and exponential phases of growth. Therefore, section “Probabilistic Individual-Based Model” starts with presenting an individual-based model that considers the life cycle of each individual cell to predict the population growth. This model is simplified until a population-based model with much lower computational load is obtained in section “Probabilistic Population-Level Model.” The parameters of the individual lag phase distribution that minimize the difference between the simulated and experimental distributions of the population size are then selected. This requires that the type of distribution of the population size is known. Therefore, several types of probability distributions will be compared in section “Population Size Distribution” to select the most suitable one. Section “Proposed Experimental and Computational Method” presents an overview of the complete experimental and computational method that is proposed. This method is validated based on simulations in section “Simulation Validation” and on experiments in section “Experimental Validation.”

### Detection Limit and Calibration Curve

Any method relying on the use of OD measurements should start with the determination of the measurement’s detection limit and making a calibration between the measured OD values and the underlying quantity of colony forming units. Based on the method explained in section “Detection Limit,” the standard deviation of the measurement was calculated to be 1.55 × 10^–3^ OD units. This corresponds with a detection limit of 4.65 × 10^–3^ OD units above the measurement blank. This detection limit includes pipetting errors because the standard deviation was determined based on replicates that were independently pipetted.

Only the seven most concentrated samples of the calibration curve had OD values above this detection limit. The remaining samples were therefore discarded from the calibration curve. The parameters *a* and *b* of the calibration curve were estimated to be 22.55 ln(CFU/mL) and 1.07 ln(CFU/mL)/ln(OD). The 95 % confidence bounds of these parameters were [22.38;22.72] and [1.02; 1.12]. The mean squared error of the regression was just 0.01 ln(CFU/mL)^2^, demonstrating a good quality of fit. Based on this calibration curve, the detection limit of 4.65 × 10^–3^ OD units corresponds with 1.98 × 10^7^ CFU/mL. This reflects the high cell density level that is required for applying OD measurements. In comparison, the detection limit for viable plate counts is generally between 10^2^ and 10^3^ CFU/mL.

### Comparison of Conventional Inoculation Methods

This section deals with the selection of an experimental method that is suitable for high throughput data collection. This method is needed because the study of the variation of the lag phase (or any other process) requires large amounts of data. As such, experimental protocol for this method needs to be selected in such a way that the quantity of useful data is maximized. In both inoculation methods, there is no guarantee of obtaining single cells in the different samples (wells of the microplate). However, the idea is to maximize the chance that single cells are obtained. The amount of useful data can be quantified by evaluating (i) the number of wells that contain cells compared to the total amount of wells and (ii) the number of wells containing exactly a single cell compared to the total number of wells containing cells.

The experimental methods that are reported, respectively, in section “Serial Dilution Inoculation Method” and section “Low Cell Density Inoculation Method” were first implemented in two sets of Monte Carlo simulation. The first set of simulation only used the Poisson distribution to determine the random number of cells that is transferred in each pipetting step. For each experimental protocol, 10^5^ iterations were run in which a 96-well microplate was inoculated with a Poisson distributed number of cells per well. In these first simulations, the number of cells in the first column was fixed to 100 for the serial dilution method. When evaluating the number of cells in the last five rows, 10.86 % of the total number of wells were found to contain cells and 77.93 % of those contained a single cell. (Table 1 part A). This latest measure was taken as a benchmark to determine the concentration of the diluted preculture for the low concentration inoculation method. As such, it was found that at a concentration of 0.48 CFU/well, the same fraction of wells with cells contained a single cell. On the other hand, from the total number of wells, almost four times as many contained cells (38.13 %). As such, the low concentration inoculation method delivers a much higher quantity of useful data for the same number of microplates and a slightly lower workload (as the serial dilutions require more effort).

**TABLE 1:** Evaluation of the serial dilution and low concentration method for the inoculation of microplate wells with single cells.

Origin of variability	Evaluation measure	Serial dilution inoculation method (%)	Low concentration inoculation method (%)
A) Cell distribution	Wells with cells	10.86 ± 5.07	38.13 ± 9.71
	Wells with 1 cell	77.93 ± 26.76	77.93 ± 13.55
B) Cell distribution, error on calibration, pipetting errors	Wells with cells	10.25 ± 5.08	37.99 ± 12.09
	Wells with 1 cell	77.93 ± 27.02	77.93 ± 14.50
C) Experimental uncertainty and microbial variability	Wells with cells	9.90	27.86
	Wells with 1 cell	84.21	82.24

*Percentages of (i) wells containing cells compared to the total number of wells and (ii) wells containing a single cell compared to the wells containing cells. **A** and **B** were calculated from Monte Carlo simulations and results are provided with 95 % confidence intervals. **C** was obtained experimentally.*

To further evaluate the difference between these methods, a second set of Monte Carlo simulation was performed. In this set of simulation, the variability that was due to the error related to the calibration curve and the pipetting errors was included as well. For these calculations, the number of cells in the first row of the microplate was no longer fixed to 200 CFU in 300 μL (of which on average half are transferred to the following wells). Instead, the model prediction error on the calibration curve was used to determine the probability distribution of the number of cells in the 1:10 diluted preculture (see calculation in section “Parameter Estimations”). Moreover, for all dilution steps, the pipetting errors were considered as well. According to the manufacturer specifications the pipetted volumes were assigned normal distributions with relative standard deviations of, respectively, 0.20, 0.25, 0.25 and 0.50 % for a single channel 200 μL pipette, a multichannel 200 μL pipette, a single channel 1,000 μL pipette and a single channel 5,000 μL pipette. As in the previous simulations, the quantity of cells in the pipetted samples followed the Poisson distribution. The resulting evaluation of the two inoculation methods is presented in Table 1 part B. These results are similar to the Monte Carlo simulation that only considered the variance due to the cell distribution over pipetted volumes. The low inoculation method is more susceptible to the effect of pipetting errors and the error on the calibration curve than the serial dilution method. This is probably because the former method relies more on the accuracy of the obtained calibration curve to perform the inoculation of the microplate at the correct cell concentration. On the other hand, the low inoculation method will always yield more wells containing cells and will have less variability on the percentage of wells that contains a single cell. As such, when considering all significant stochastic processes in these methods, it was found that the low inoculation method yielded the highest amount of useful experimental data.

To prove the conclusions of the simulation study, an experimental validation was performed on four independent biological replicates for each protocol. This resulted in 384 inoculated wells for each inoculation method. The evaluation of the experimental results is presented in Table 1 part C. Compared to the simulation studies, there was a lower yield of wells containing cells and a higher percentage of wells containing a single cell. This was found to be due to having a lower inoculation in practice, than was calculated from the calibration curve. Irrespective of this, the experimental results are in line with the Monte Carlo simulation and confirm the conclusion that the low concentration inoculation method delivers more useful experimental data. Moreover, the experimental work demonstrated that the low inoculation method required the same amount of lab consumables and had a slightly lower workload. Based on these results, the low concentration inoculation method was selected to be most suitable as part of an efficient and accurate method to determine single cell lag.

### Probabilistic Individual-Based Model

**FIGURE 1:**
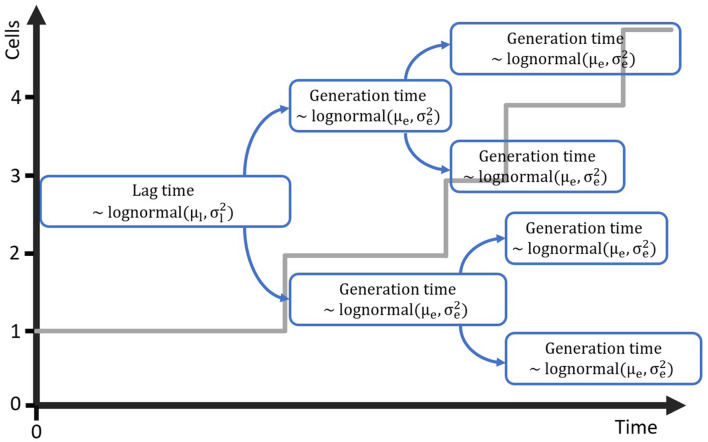
Probabilistic simulation of the growth of a population starting from a single cell with generation time in the lag phase distributed according to the lognormal distribution with parameters μ_**l**_ and $\sigma_{l}^{2}$. Each of the daughter cells is assigned a generation time for the exponential phase from the lognormal distribution with parameters **μ_e_** and $\sigma_{e}^{2}$.

The computational method that will be developed requires a mathematical model that is suitable for simulating cell growth and its variability, starting from a single cell. Individual-based models are perfectly suitable for this task, as they describe every cell individual and the variation between these cells can be included in that description. As such, the next step in the method development is to propose and test a very simple individual-based model that can be used to simulate the lag phase and growth starting from a single or few cells. The first step in this process was to create a probabilistic simulation of the growth of a population starting from a single cell. This simulation is illustrated in Figure 1. The simulation assumes that cells have a specific distribution for their generation time in the lag phase and the exponential phase. The lag phase is considered to be the time until the first division after a cell enters a new environment. This assumption is supported by the research of Pin and Baranyi (2006) in which individual cell division times were observed through microscopy experiments. For a specific set of experiments with *E. coli* K12, the time for the first, second, third and fourth division were found to be 3.30, 1.04, 0.98 and 0.92 h. This demonstrates that the lag phase duration is mainly related to the time until the first division for single cells. For the current paper, this time until first division is taken equal to the individual lag time. It should be noted that some researchers consider the time until the first division to be the sum of the lag time and the generation time. When the generation time is known, a simple conversion between the two definitions is possible. To create a simulation case study for the model development, the distribution parameters for the lag phase duration and generation time were based on the data of Pin and Baranyi (2006). The individual lag phase duration was assumed to have a mean of 3.30 h and standard deviation of 1.02 h, and the generation time a mean of 0.92 h and standard deviation of 0.29 h. Given that the lag and generation time can never be negative, these growth parameters cannot be described by a normal distribution and a lognormal distribution was assumed for these processes (Koch, 1966). In the past, researchers have also used other distributions such as the Weibull, gamma and exponential distribution to describe individual cell lag (Standaert et al., 2007). It is important to stress that the results from this study would not be different if a different probability distribution for the individual cell lag was assumed.

**FIGURE 2:**
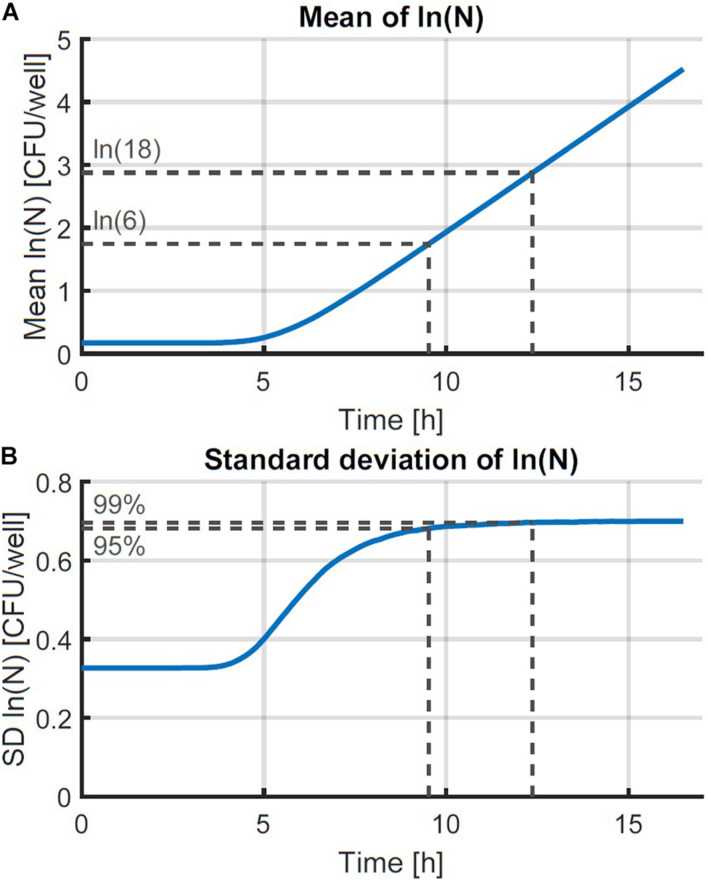
Monte Carlo simulation of the growth of small microbial populations with random lag time and exponential generation time. The mean **(A)** and standard deviation (SD) **(B)** of the obtained populations are shown as a function of time. The striped lines indicate the point at which 95 and 99 % of the maximum variability are reached at 6 and 18 CFU, respectively.

The individual-based simulation starts from a single cell that is assigned a lag time from the lognormal distribution of the individual lag phase. Once this timepoint is reached, the cell is replaced by two daughter cells. Each of these daughter cells is assigned a random generation time according to the lognormal distribution for the generation time in the exponential phase of growth. When these daughter cells reach their individually assigned generation times they are replaced in the same manner and so on. This simple simulation can also be made by starting from a set of multiple cells, each with a randomly assigned individual lag time. Following this random process, it is, however, not possible to estimate the parameters from both the distribution of the individual lag time and the generation time because they would be highly correlated. Therefore, it is desirable that these two probability distributions can be simplified to a single distribution. This simplification is only possible if the model with a single probability distribution can accurately describe the variability of the process. To test this possibility, a Monte Carlo simulation with 10^5^ iterations was performed. Each iteration simulated the inoculation and growth of a microorganism in the well of a microplate, according to the low concentration inoculation method established in section “Comparison of Conventional Inoculation Methods” (0.48 CFU/well). In each well containing cells, the growth occurred according to the individual-based simulation with random distributions for both the individual lag time and exponential generation time. After obtaining all growth curves, they were transformed to a logarithmic scale and the mean and standard deviation were calculated as a function of time (Figure 2). The evolution of the standard deviation of the population size shows a sigmoidal behavior. This is because once the population reaches a certain size, the fast and slow growing cells will balance each other out and different populations all grow at approximately the same rate. As such, when a certain population size is reached, there is no further evolution of the variability of the population growth on a logarithmic scale.

The evaluation of the variability was further assessed by defining the population size where the variability settled to a near-constant value. The Monte Carlo simulations were carried out in parallel for increasing time values and the simulation was stopped at the point where the relative increase of the standard deviation of the population size was less than 0.01%. As such, the final standard deviation of the population size can be taken equal to the maximum standard deviation that could be reached. Then, the population sizes were calculated at which 95 and 99 % of the increase from the initial to the final standard deviation were achieved. As such, it was found that under the simulation conditions, the variability of the population size settled to a constant value between population sizes of just 6–18 CFU on average. Although these values depend on the simulation parameters, they illustrate that the variability reaches an equilibrium at very low population sizes. This is in agreement with the results of, e.g., Kutalik et al. (2005) who found the standard deviation of the division time to decrease after the first division.

These results demonstrate that the probabilistic simulation model can be simplified from a model that has separate distributions for the individual lag time and exponential generation time to a model that only has a distribution for the individual lag time and a constant generation time in the exponential phase. This simplification holds under the assumption that the model will be used to describe the variability on the population size after a few generations. As such, the variability of the initial phases of population growth can be studied by analyzing the variability on the population size after just a few generations.

### Probabilistic Population-Level Model

The simulations with the individual-based model of section “Probabilistic Individual-Based Model” provide a very accurate description of the variation of population growth. However, given the algorithm that needs to calculated with a high number of iterations, simulating this model is slow. To allow the proposed method to work, a model should be constructed that approximates the individual-based model but can be computed much faster. The model will be incorporated as part of a Monte Carlo method within a parameter estimation and will therefore be computed a high number of times. Therefore, a population-level model should be constructed that can be expressed by a simple mathematical expression. The following simple mathematical expression is proposed for the growth of a population starting from a single cell:


(3) $$\ln\left({\text{N}}_{\text{s}}\left(t\right)\right)=1\left(t-\lambda_{\text{i}}\right)\cdot \mu \cdot \left(t-\lambda_{\text{i}}\right)$$

with N_s_ [CFU] the number of cells originating form a single cell, t [h] the time, _i_ [h] the individual lag time and [h^–1^] the maximum specific growth rate in the exponential phase of growth. The function 1(⋅) represents the unit step function, which is 0 for all arguments smaller than 0 and 1 for all arguments greater or equal than 0. As such, Equation 3 remains constant at 0 during the individual lag time (*t*_i_) and predicts log-linear growth for all times larger than _i_. For populations, N, starting from multiple cells, N_0_, the equation can be written as:


(4) $$\ln\left(N\left(t\right)\right)=\ln\left({\text{N}}_{0}\right)\sum_{i=0}^{N_{0}}1\left(t-\lambda_{i}\right)\cdot \mu \cdot \left(t-\lambda_{i}\right)$$

**FIGURE 3:**
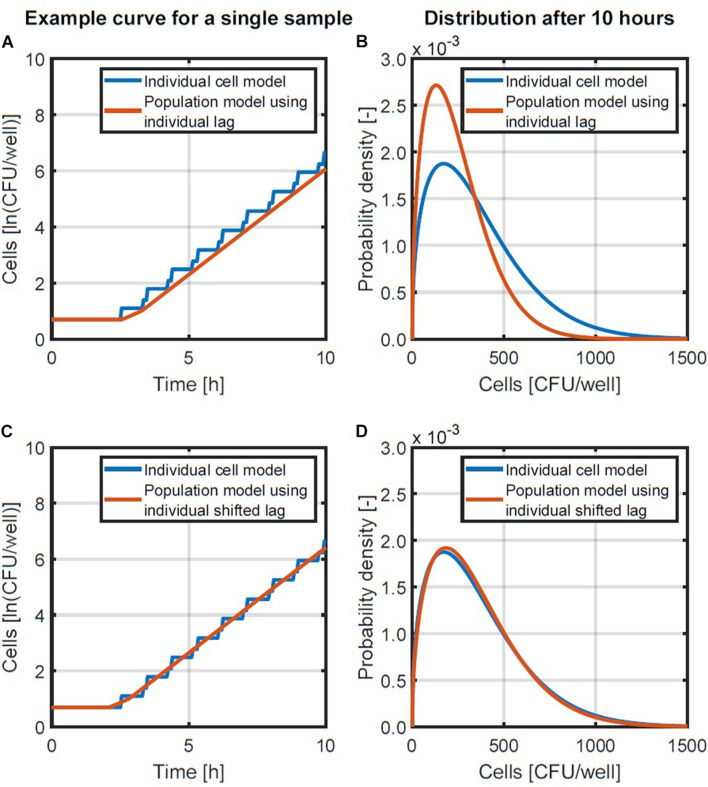
Examples of the individual-based and population-level models **(A,C)** and the probability density functions of population sizes after 10 h **(B,D)**. A comparison is made between a population-level model using the individual lag time directly **(A,B)** and a population-level model in which the lag time is reduced with half of the generation time **(C,D)**.

The summation is used in this equation since the individual lag times are not equal but obtained from a random distribution. The population-level model in Equation 4 was compared with the individual-based model with probability distribution for the individual lag time and fixed generation times. This comparison was done by performing a Monte Carlo simulation with 10^5^ iterations and the same parameters for the lag time distribution as the simulation in section “Probabilistic Individual-Based Model.” The simulation results are presented in Figures 3A,B. Figure 3A compares an example simulation from the two different models for the same initialization parameters (initial number of cells and their individual lag times). Figure 3B provides the lognormal probability density functions that were fitted to the population size data after 10 h, when the variability on the population size has long settled. As can be seen from these results, there is a discrepancy between the obtained probability distributions. This discrepancy in the probability distributions was because the predicted population size with the population-level model was always lower than that of the individual-based model (Figure 3A). This difference in the predicted growth curves of each model originates from the stepwise evolution of the individual-based model whereas the population-level model lags behind with a gradual increase that starts at the end of the lag time. This difference can be balanced out by shortening the lag phase duration in the population-level model by half the generation time in the exponential phase. This correction essentially applies a time shift to the exponential phase so that the log-linear increase ends up halfway of the stepwise increase (see Figures 3A,C). Since the generation time can be expressed as ln(2)/, the model in Equation 4 is than converted to the following expression:


(5) $$\begin{array}{c} \ln\left(N\left(t\right)\right)=\\ \ln\left({\text{N}}_{0}\right)+\sum_{i=1}^{N_{0}}1\left(t-\lambda_{i}+\frac{\ln\left(2\right)}{2\mu }\right)\cdot \mu \cdot \left(t-\lambda_{i}+\frac{\ln\left(2\right)}{2\mu }\right) \end{array}$$

A comparison of the population model in Equation 5 and the individual-based model is presented in Figures 3C,D. Figure 3C demonstrates with an example that the new population model is a linear approximation of the stepwise behavior of the individual-based model. Figure 3D illustrates that this change in the population model results in a good approximation of the distribution of the population size that is obtained. As such, the model in Equation 5 will be used to simulate the distribution of the population density.

Without proving this point, it is worth mentioning that it is possible to calculate the final population size, as done in Equation 5, by first calculating the population lag, _p_, using the following equation:


(6) $$\lambda_{\text{p}}=-\frac{1}{\mu }\cdot \ln\left(\frac{1}{N_{0}}\sum_{i=1}^{N_{0}}\text{exp}\left(\frac{\ln\left(2\right)}{2\mu }+\lambda_{i}\right)\cdot \mu \right)$$

The mean population density is then calculated as:


(7) $$\ln\left(N\left(t\right)\right)=\ln\left({\text{N}}_{0}\right)+1\left(t-\lambda_{\text{p}}\right)\cdot \mu$$

Although seemingly simpler, the fact that the summation remained necessary through Equation 6, meant that the computational load did not decrease in the current study. As such, all calculations of the population size were carried out with Equation 5 as a population model.

### Population Size Distribution

Now that the population-based model has been proposed, all that remains to finalize the method is to find the probability density function that is most suitable to describe the population size at a given time point. This probability density function is required within the parameter estimation to compare the simulated model output with the experimentally measured probability distribution of the population size. The optimal parameters of the distribution of the individual cell lag are those parameters that minimize the difference between the simulated and experimental distributions of the population size.

**FIGURE 4:**
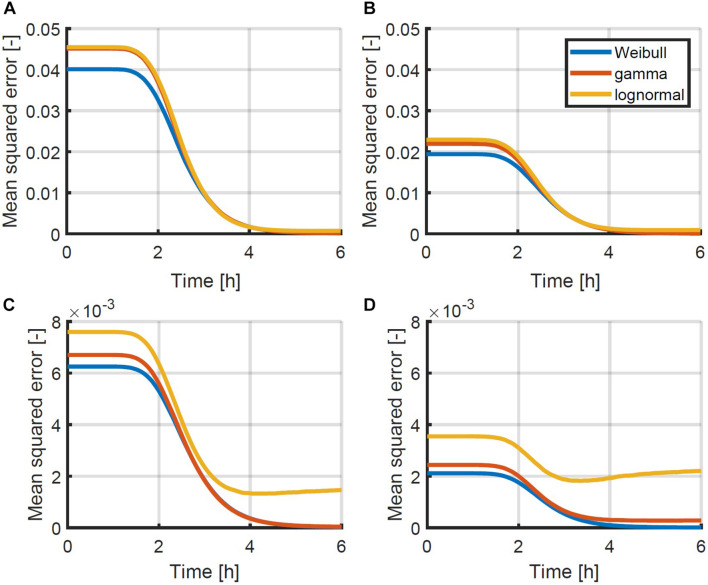
Mean squared error between the simulated cumulative distribution of the population size as a function of time and the approximated Weibull, gamma and lognormal distribution for initial population sizes: **(A)** 0.5, **(B)** 1.0, **(C)** 2.0, and **(D)** 4.0 CFU/well. Simulations were performed using the individual-based model of Figure 1 with the parameters described in section “Probabilistic Individual-Based Model”.

To test which probability density function would be most suitable, a set of simulation data was generated. The data was generated for 1,000 96-well plates, inoculated at intended concentrations of 0.5, 1.0, 2.0 and 4.0 CFU/well according to a Poisson distribution. In each sample containing cells, the population growth was simulated for a period of 6 h, using the individual-based model of Figure 1 with the parameters of Pin and Baranyi (2006) as mentioned in section “Probabilistic Individual-Based Model.” At each time point, the Weibull, gamma and lognormal distribution were fitted to the distribution of the population size. The mean squared errors between the data and the distributions were calculated based on the cumulative distributions and plotted as a function of time in Figure 4. As can be seen from this figure, the Weibull distribution achieves a better approximation of the distribution of the population size for all inoculum sizes and at all time points. As such, the Weibull distribution was selected as the most suitable distribution to describe the population size and was used within the following parameter estimations to determine the individual lag phase distribution. The small differences between the approximation by the different distributions is in line with the results of Huang (2016) who found little difference between simulation results using the normal, lognormal, Gumbel, gamma, Weibull, and exponential distributions.

### Proposed Experimental and Computational Method

The current section describes all experimental and computational steps that are proposed in the current method for the estimation of the individual lag time distribution. The following steps are taken: (i) determine the OD detection limit and calibration curve between OD and colony forming units, (ii) perform a growth experiment to find the growth rate in the exponential phase, (iii) inoculate microplates with several cells per well and quantify the number of cells after a period of time, (iv) correct all measurements to the exact same sampling time and (v) perform a parameter estimation to find the distribution of the individual lag time that results in the same distribution of the cells at the sampling time.

Even though the proposed method relies primarily on the use of viable plate counts, OD measurements are a useful tool for the inoculation procedure. The availability of a calibration curve between the OD and the number of viable plate counts allows the estimation of the cell density of the preculture or one of its dilutions. This cell density should be known for the calculation of the desired dilutions to start the actual experiments. Since the cell density of the preculture will often vary, the OD can provide a fast measurement of the cell density before starting the inoculation. The detection limit is needed to know the lower bound of the calibration curve. If it is not specifically desired to aim at the same initial cell density among replicates, this step can also be omitted. The initial concentration needs to be verified in any case based on plate counts for a more accurate determination.

The next experimental and computational steps require knowledge on the growth of the microorganism under the relevant conditions. To this end, a growth curve is constructed under the same conditions as during the actual experiments. In the current research, the growth curve is therefore also constructed using the same 96-well plates. Once the growth curve is available, a parameter estimation with the simple three-phase linear model (Equation 2) will provide the exponential growth rate which is essential information for the computational part.

The next step is to inoculate samples with a relatively low quantity of cells. The idea is to have a high chance of obtaining at least one cell in every sample while not exaggerating with the number of cells per sample either. After all, too high a number of cells could cause the cells to influence each other during the first stages of the microbial growth, which is not desirable in this type of experiments. Before inoculating the samples, it is also important to determine the concentration of the inoculum using viable plate counts. After a certain period of time, the quantity of cells in each sample is determined using the viable plate count method. The fastest and most accurate method is to transfer the entire sample volume to petri plates. This is possible when using small sample sizes (e.g., 100 μL) and if the total number of cells in the sample is kept limited (e.g., maximum 300 cells). As such, the time point of sampling should be selected in such a way that the individual lag phase has most likely passed but the number of cells has not yet increased by too much. The growth experiment from the previous step will indicate the appropriate time point.

The fact that the current method relies on plating the entire undiluted volume of each sample, yields a relatively fast experimental method. That is to say, samples from a full 96-well plate can be transferred directly to 96 agar plates. However, compared to OD methods, it is important to understand that there is a significant additional work load involved. Obtaining an OD measurement in a microplate reader takes only a few minutes. On the other hand, even this efficient protocol for plate counts would take at least about 1 h and could extend to several hours, depending on the approach. The method can be done in less than an hour when using ready-to-use agar plates in combination with an automatic colony counter. When using self-prepared agar plates and performing manual plate counts, the method requires at least 2 h for a single 96-well plate.

After incubating the petri plates and counting colonies, the final number of cells in each sample is known. However, depending on the number of samples, there can be a significant difference in the time between inoculation and sampling. As such, it is important to determine the time *t* between inoculation and sampling for each sample. All sample quantities can then be recalculated for the mean sampling time $\bar{t}$. The logarithm of the sample quantity *n* [ln(CFU)] is then converted to $n-\mu \cdot \left(t-\bar{t}\right)$, with μ being the previously estimated exponential growth rate. After this correction, a distribution of the cell population size is obtained at a specific time point $\bar{t}$.

At this point, the following is known: (i) the distribution of the initial quantity of cells at the time of inoculation, which follows a Poisson distribution with the average number of cells per sample as its only parameter, (ii) the exponential growth rate μ, (iii) the time of sampling $\bar{t}$ and (iv) the distribution of the population size at that point in time. It should be noted that the Poisson distribution of the initial quantity of cells is valid in this case because the experimental method relies on the distribution of samples from a single suspension with a random distribution of bacteria. If the experimental method were to be changed, it should be considered if this distribution needs to be changed as well. It is also possible to simulate a distribution of the population size by using the population-based model of Equation 5 in a Monte Carlo simulation with randomly generated initial cell quantities from the known Poisson distribution and randomly generated individual lag phase durations from a the lognormal distribution. The parameters of this lognormal distribution are, however, unknown. These parameters can therefore be found by selecting them in such a way that the simulated and measured distributions of the population size approximate each other best. To quantify the distance between both probability distributions, a Weibull distribution is fitted to the simulated distribution and the difference between the cumulative form of that Weibull distribution and the cumulative probability density of the data is calculated. Finding the parameters of the lognormal distribution of the individual lag phase can be achieved by solving this optimization problem with the function *lsqnonlin* of MATLAB.

### Simulation Validation

Methods to determine the variation of a lag phase distribution are difficult to validate because testing the reliability of these methods requires high amounts of experimental data. Therefore, the first step in validating the proposed method is to validate based on the theoretical simulations. These simulations allow the repeated calculation of a simulated experiment to test the reproducibility of the results. To obtain representative simulations, the same steps are followed that would be applied in an experimental study. This validation was carried out using these steps: (i) a first dataset was generated to estimate the growth rate, (ii) two datasets were generated at different initial concentrations to determine the lag phase distribution by using the individual-based growth model, (iii) the lag phase distribution was estimated for both datasets, (iv) a Monte Carlo simulation of the population growth was made using the population-based model and estimated parameters, and (v) the results were compared with those of a Monte Carlo simulation of the original individual-based model.

**FIGURE 5:**
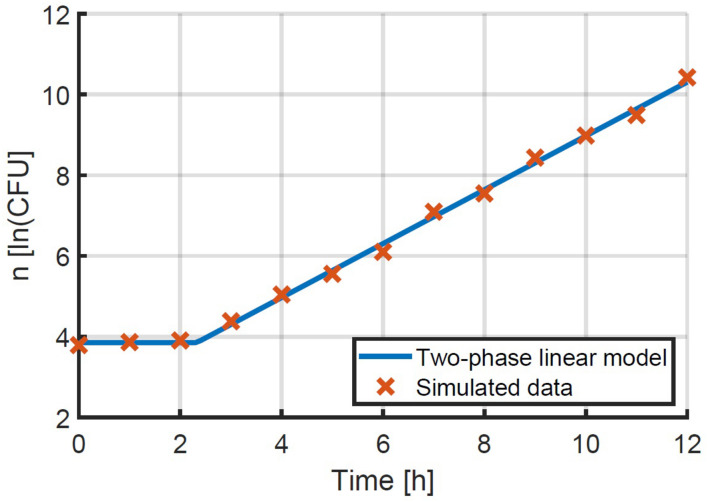
Simulated data starting at Poisson distributed initial concentrations and following growth according to the individual-based model of Figure 1. The two-phase linear model was fitted to this data (Equation 2).

When following the proposed method, the first step is the estimation of the microbial growth rate. This step was simulated by generating inoculation data for a 96-well plate with liquid samples of approximately 48 CFU/well according to a Poisson distribution. For each sample, the growth was simulated according to the individual-based model that is described in Figure 1. The mean and standard deviation for the generation time of both the lag phase and exponential growth were again taken from Pin and Baranyi (2006) to obtain realistic simulations: 3.30 and 1.02 h, and 0.92 and 0.29 h respectively. The growth of all samples was simulated for a period of 12 h. Three random samples were taken every hour out of the simulated results and their averages were calculated as a function of time. These data were used to estimate the lag phase duration and exponential growth rate by fitting the three-phase linear model (Equation 2) to the simulated data (Figure 5). The lag phase duration of the population was found to be 2.24 h and the exponential growth rate was 0.834 1/h. This exponential growth rate is essential to determine the lag phase distribution in the following step.

In the second step, two datasets were simulated, each consisting of 4 96-well plates. These datasets were inoculated with intended concentrations of 0.48 and 4.80 CFU/well according to a Poisson distribution. In all wells containing cells, growth was simulated according to the same individual-based model to obtain the population size after 6 h. These simulated data correspond to the experimental data that would be obtained when determining the viable plate counts of each sample after a given time. As such, these data were used as inputs for two parameter estimation problems to estimate the parameters of the lognormal distribution that describes the individual lag phase duration. The mean and standard deviation of this distribution were calculated to be, respectively, 3.17 (3.14; 3.20) and 1.18 (1.14; 1.23) h, and 3.27 (3.25; 3.28) and 1.12 (1.08; 1.17) h for initial population sizes of 0.48 and 4.80 CFU/well. These parameter values are close to the mean and standard deviation of the individual lag phase duration that were used in the individual-based model to generate the data (3.30 and 1.02 h). It can be noted that the standard deviations were found to be higher than that specified to run the simulation with the individual-based model. This is due to the fact that the standard deviation of the individual lag phase duration in the population-based model captures the variability of both the lag phase duration and the generation time in the exponential phase. The small difference between the parameters used to simulate the data and the estimated parameters is already a first indication of the successful implementation of the proposed method.

Baranyi et al. (2009) have published a methodology that is based on the method of moments for calculating the individual lag phase distribution from the same type of data as described here. This method has a much lower computational burden than the method proposed in this work, which requires solving a maximum likelihood estimation problem that involves a Monte Carlo simulation. As such, this moments-based method was compared on exactly the same simulation data. To this end, the mathematical formulation to solve this problem was taken directly from Baranyi et al. (2009). For the data at 4.80 CFU/well, a decent estimate of the individual lag phase distribution was obtained, with a mean of 2.90 and standard deviation of 1.16 CFU/well. However, no reasonable solution could be obtained for the low concentration data of 0.48 CFU/well. Upon further examination, it was found that the accuracy of the estimated distribution decreased with decreasing initial concentrations. Combining this inaccuracy with the fact that this method provides no uncertainty on the parameter estimates, the moments-based method was found to be unsuitable for solving the current problem.

**FIGURE 6:**
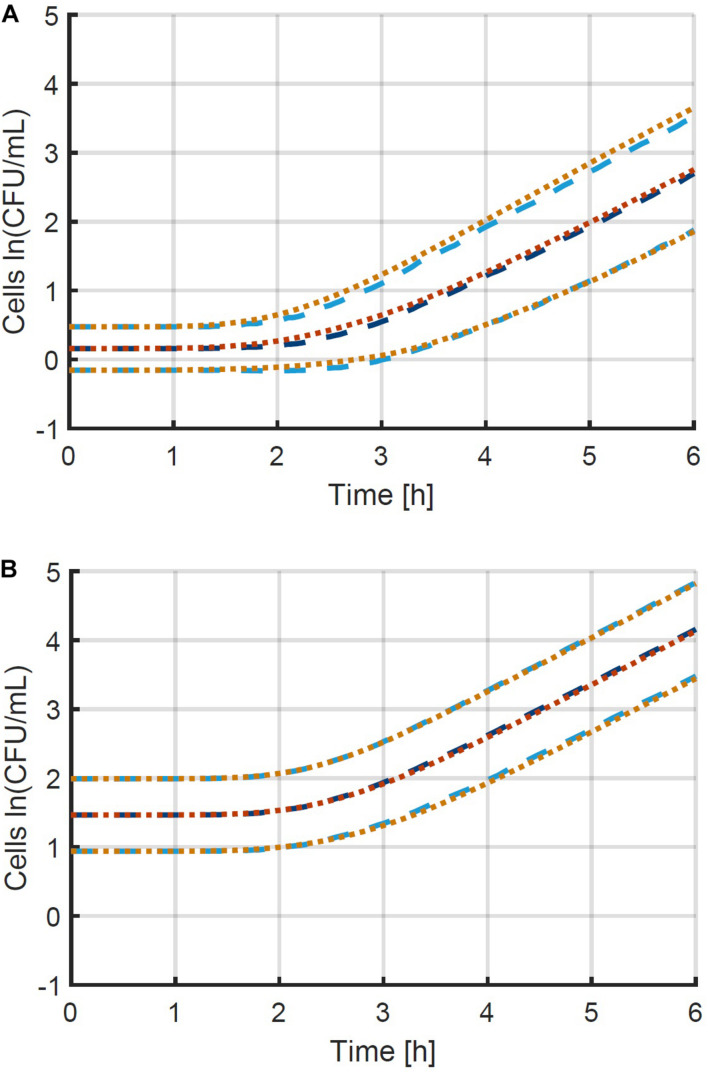
Comparison between the mean and standard deviation of the population size according to (i) the individual-based model used to generate simulation data (— and …) and (ii) the population-based model that approximates this data (— and …). This information is represented for the two case studies with initial population sizes according to a Poisson distribution with means of **(A)** 0.48 and **(B)** 4.80 CFU/well. The individual-based model and parameters are described in section “Probabilistic Individual-Based Model”.

A more thorough validation of the obtained method and parameter estimation results was done by comparing a simulation of the individual-based model using the original parameters with a simulation of the population-based model using the estimated parameters. The simulations were performed for 10 96-well plates at the original average inoculation densities and the resulting mean and standard deviation of the population size as a function of time are presented in Figure 6. The number of plates (iterations) was chosen to obtain an accurate estimation of the standard deviation as a function of time, as indicated by smooth curves. Comparing the two model simulations in the figure demonstrates that the newly developed method enables the population-based model to provide a very good approximation of both the average population behavior and the individual cell variability. Specifically, the ability of the method and the population-based model to describe the variability is remarkable. Even though the model completely ignores the variability between individual cells in the exponential phase of growth, a good approximation is achieved, starting from the initial concentration of cells, all the way into the exponential phase of growth. This validation study proves that it is indeed possible to capture the combined variability of the cells in the lag and exponential phase of growth into a single variable, being the individual lag phase duration. The prerequisites to obtain the current results are that both the mean initial population size and the exponential growth rate are determined accurately. The final step in the method development is the experimental validation.

### Experimental Validation

**FIGURE 7:**
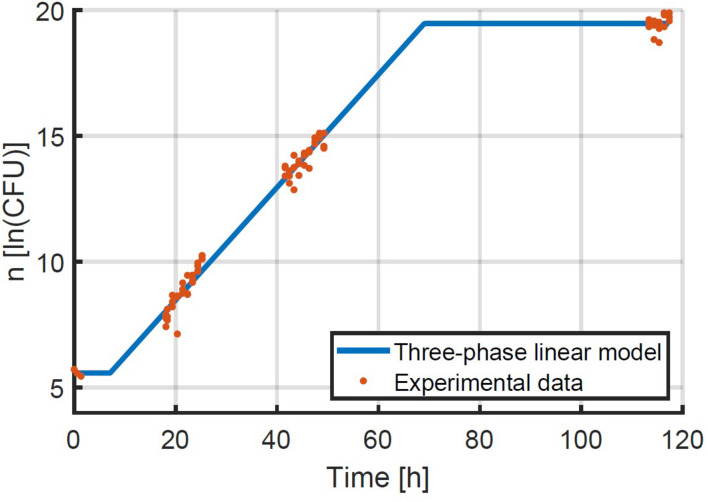
Data on the growth of *E. coli* at 25°C after being transferred to BHI medium with 56 g/L NaCl at an average quantity of 48 CFU/well. The three-phase linear model of Equation 2 was fitted to the data.

Given that the simulation validation was successful, an experimental validation was performed specifically to test the assumption that the method can be used at higher inoculation levels and therefore at higher yields of experimental data. The method for the experimental validation study is explained in detail in section “Experimental Study of the Population Variability.” The first step in the evaluation of the experimental data was to estimate the exponential growth rate based on the experiments that were inoculated at an intended concentration or 48 CFU/well. The exponential growth rate μ was estimated to be 0.224 1/h with 95 % confidence bounds of 0.216–0.232 1/h. This experimental data and the fitted model are illustrated in Figure 7. The 95 % confidence bounds indicate a high level of confidence in the estimated growth rate, because of the large number of samples. The lag phase duration was estimated to be 7.10 h (4.88; 9.33).

The next step was to process the data on the distribution of the population size. Given the difference in inoculation and sampling times between all samples, it was considered that not all samples were taken after the same period of time. The average sampling time $\bar{t}$ was calculated for all samples and the logarithm of the sample quantity *n* was corrected to $n-\mu \cdot \left(t-\bar{t}\right)$. In this manner, all sample quantities were obtained at the exact same time point, which was 26.79 h for the experiments at 0.48 CFU/well and 21.97 h for the experiments at 4.80 CFU/well. Based on the quantification of the concentration of the diluted inoculum, the real average initial concentrations were calculated to be 0.35 and 3.07 CFU/well. Based on the known exponential growth rate, sampling time and initial concentrations, the parameter estimation to determine the distribution of the individual lag phase duration could be performed as explained in section “Proposed Experimental and Computational Method.” These parameter estimations led to the identification of the mean and standard deviation of the lognormal distribution of the individual lag phase duration with 95 % confidence bounds to be 9.72 (9.65; 9.79) and 1.77 (1.62; 1.93), and 9.80 (9.78; 9.82) and 1.95 (1.90; 2.00) for the lower and higher inoculation level, respectively. These parameter estimates were in good agreement between the experiments at different inoculum levels and they were relatively accurate. The parameter accuracy of the experiments started at the low inoculum level was lower than those started at a high level. This is simply due to the much lower number of wells that contained cells for the low inoculum level (95 wells compared to 375 wells). As such, these experiments demonstrated first of all that the characterization of the individual lag phase duration is possible with the current method at various inoculum levels. More importantly even, these experiments have shown that this new method allows for the determination of the individual lag phase variability with the same precision, while requiring a much lower experimental effort by starting the experiments at inoculum levels that are more likely to place at least one cell in every well instead of aiming at a high chance of having no more than 1 cell per well.

A final step in this validation was to predict the lag phase duration for the experiment with the highest inoculum level that was used to estimate the exponential growth rate, based on the estimated distributions of the individual lag phase. This was done using the population-based model at the same inoculum level of 39.76 CFU/well for 1,000 iteration. The lag phase durations were estimated by approximating the simulation data with the two-phase linear model. Based on the distribution from the inoculum of 0.35 CFU/well the lag phase was estimated to be 7.68 h and for the distribution from the inoculum of 3.07 CFU/well it was 7.61 h. The predicted lag phase durations were close to each other because of the probability distributions being very similar. Moreover, the predicted population lag times were in line with the lag phase duration of 7.10 h that was estimated from the growth data at the highest inoculum level. Both the data and simulations demonstrated a reduction of the population lag phase duration with increasing population size. This reduction of the lag phase duration can be understood from the larger likelihood of having fast growing cells that will dominate the population lag. Moreover, this phenomenon has been demonstrated in previous research. For example, Robinson et al. (2001) demonstrated this effect experimentally for *L. monocytogenes* at increased NaCl concentrations.

## Conclusion

This research presents a combined experimental and computational method for the determination of the individual lag phase variability. The experimental method can readily be implemented in most microbiology labs and the computational combines robust techniques such as Monte Carlo simulation and maximum likelihood parameter estimation, which are familiar to researchers in the domains of predictive microbiology and quantitative microbial risk assessment. The experimental method requires an accurate estimation of the average initial concentration and the growth rate under the given conditions. By demonstrating that the variability on the logarithmic concentration of the population settles after just a few generations, it is possible to quantify this variability of the population size as a measure for the variability on the initial growth phases. The further simulations demonstrated that a simple population-based model can accurately approximate and predict this growth variability as a consequence of individual cell variability. The experimental validation demonstrated the ability of the proposed methodology to determine the probability distribution of the individual lag phase through experiments at higher inoculum levels (>1 CFU/sample). The advantage of these higher inoculum levels is that a larger amount of useful data (wells containing cells) can be obtained from the same experimental effort and cost. Moreover, by plating full undiluted samples in the experimental method, the experimental error is reduced to a minimum. As such, the proposed method delivers high accuracy while minimizing the experimental load.

## Data Availability Statement

The raw data supporting the conclusions of this article will be made available by the authors, without undue reservation.

## Author Contributions

SA performed the experimental and computational work. SA and JV coordinated the research and contributed to the writing. Both authors contributed to the article and approved the submitted version.

## Conflict of Interest

The authors declare that the research was conducted in the absence of any commercial or financial relationships that could be construed as a potential conflict of interest.

## Publisher’s Note

All claims expressed in this article are solely those of the authors and do not necessarily represent those of their affiliated organizations, or those of the publisher, the editors and the reviewers. Any product that may be evaluated in this article, or claim that may be made by its manufacturer, is not guaranteed or endorsed by the publisher.
